# Endothelial Nitric Oxide Synthase-Dependent Mechanism of Hydroxyurea-Induced S-Phase Arrest in Erythroid Cells

**DOI:** 10.3390/antiox15040435

**Published:** 2026-03-31

**Authors:** Teodora Dragojević, Dragoslava Đikić, Slavko Mojsilović, Miloš Lazarević, Dejan Milenković, Olivera Mitrović Ajtić, Emilija Živković, Miloš Diklić, Tijana Subotički, Juan F. Santibanez, Vladan P. Čokić, Milica Vukotić

**Affiliations:** 1 Department of Molecular Oncology, Institute for Medical Research, National Institute of the Republic of Serbia, University of Belgrade, 11029 Belgrade, Serbia; teodora.dragojevic@imi.bg.ac.rs (T.D.); dragoslava@imi.bg.ac.rs (D.Đ.); oliveram@imi.bg.ac.rs (O.M.A.); emilija.zivkovic@imi.bg.ac.rs (E.Ž.); milos.diklic@imi.bg.ac.rs (M.D.); tijana@imi.bg.ac.rs (T.S.); jfsantibanez@imi.bg.ac.rs (J.F.S.); vl@imi.bg.ac.rs (V.P.Č.); 2 Department of Hematology and Stem Cells, Institute for Medical Research, National Institute of the Republic of Serbia, University of Belgrade, 11029 Belgrade, Serbia; slavko@imi.bg.ac.rs; 3 Department for Human Genetics, Implantology Research Center, Faculty of Dentistry, University of Belgrade, 11000 Belgrade, Serbia; milos.lazarevic@stomf.bg.ac.rs; 4 Institute for Information Technologies, University of Kragujevac, 34000 Kragujevac, Serbia; dejanm@uni.kg.ac.rs

**Keywords:** hydroxyurea, nitric oxide synthase, replicative stress, proliferation, apoptosis

## Abstract

Hydroxyurea (HU) is a ribonucleotide reductase inhibitor widely used for the treatment of sickle cell disease and myeloproliferative disorders, yet a precise nitric oxide (NO) synthase (NOS)-dependent mechanism remains incompletely defined. The role of NOS3 in HU-mediated proliferation, cell cycle, and apoptosis was analyzed in HEL92.1.7 erythroleukemic cells and primary mouse erythroid progenitors upon genetic knockdown/knockout and pharmacological NOS2/NOS3 inhibition. NOS3 expression, phosphorylation, NO and citrulline production, and protein nitrosylation were assessed via immunoblotting and biochemical assays. Computational docking and molecular dynamics simulations were performed to examine the interaction between HU and NOS3. HU enhanced NOS3 expression and phosphorylation, leading to increased NO and citrulline production. Computational analysis predicted HU binding within the NOS3 active site, whereas functional activation was AKT1-dependent. A biotin switch assay revealed cooperative NOS2-/NOS3-mediated protein nitrosylation under HU treatment. NOS3 depletion or inhibition abrogated HU-induced S-phase accumulation and restored cell proliferation. NOS3 protein depletion increased late apoptosis in erythroleukemic cells, while in murine erythroid cells, both Nos3 deficiency and inhibition decreased early and increased late apoptosis. NOS2 and NOS3 act as complementary mediators of proliferation and apoptosis, with NOS3 playing a distinct role in HU-induced proliferation arrest in erythroid cells. These findings highlight the therapeutic potential of NOS targeting to enhance the efficacy of HU and overcome resistance in hematologic malignancies.

## 1. Introduction

Hydroxyurea (HU) is a well-known inhibitor of ribonucleotide reductase and is commonly used in the treatment of sickle cell disease and myeloproliferative neoplasms [1,2]. HU increases the bioavailability of nitric oxide (NO), adding to its therapeutic effect [3,4,5]. Several studies have reported the NOS-independent effects of HU; for example, it raised nitrite levels in red blood cells (RBCs) despite decreased NOS activity [3]. In addition, both HU and L-arginine boost the production of nitrite and nitrate (NOx) in RBCs, which is not affected by the NOS inhibitor N(G)-monomethyl-L-arginine (L-NMMA) [6]. In human erythroid progenitors and K562 erythroleukemic cells, HU-induced NOx accumulation is enhanced when combined with L-NMMA, but not when combined with pan-NOS inhibitors like NG-nitro-L-arginine-methyl ester (L-NAME) and NG-nitro-L-arginine (L-NNA), indicating differential NOS isoform involvement [7].

Despite these findings, other studies support NOS-dependent mechanisms of HU-induced NO production. HU elevates plasma nitrite levels and increases the expression of endothelial NOS (NOS3) in the renal vasculature in humanized sickle cell mice [8] and endothelial cells [9] while raising L-citrulline production in rats [10]. In the presence of human RBCs or heme, HU stimulates NO production in endothelial cells, where this stimulation is prevented by L-NAME [9,11]. However, HU increases endothelial cell production of NO through cAMP-dependent protein kinase (PKA) and RAC-alpha serine/threonine protein kinase (AKT) signaling, which is blocked by L-NAME and L-NNA [12]. HU also induces phosphorylation of NOS3 via PKA and AKT signaling while increasing NOS3 protein levels by inhibiting proteasome activity [12,13]. HU elevates calcium levels in both RBCs and endothelial cells, which are necessary for constitutive NOS activation [12,14]. In a previous study, L-NAME prevented HU-induced apoptosis in erythroleukemic K562 cells and hypocellularity in murine bone marrow [15]. The myelosuppressive effects of HU were reversed by the pan-NOS inhibitor L-NAME, as observed in rescued late erythroid progenitors [15]. The reported competitive and non-specific NOS inhibitors primarily target the enzyme’s L-arginine binding site, but they do not distinguish between NOS1-3 isoforms [16].

To address the conflicting research findings and better define the specific roles of NOS isoforms in the effects of HU, our previous study demonstrated individual inducible NOS (NOS2) involvement in erythroid cell proliferation and apoptosis [17]. Building on these findings, in this study, we focus specifically on the NOS3 isoform and the dual role of NOS2/NOS3 in the molecular mechanism of HU action. By employing a combination of in silico molecular docking and dynamics simulations, in vitro enzymatic assays, and cell-based studies using NOS3 knockdown and the NOS3 inhibitor Caveolin-1 scaffolding domain peptide (CSD) in HEL92.1.7 erythroleukemic cells, along with in vivo validation in Nos3-deficient mice, we demonstrate a role of NOS3 in HU-driven regulation of cell cycle and apoptosis. To observe the combined participation of NOS2 and NOS3 in the cell cycle and apoptosis regulation under HU stress, an additional dual inhibitor is used in erythroleukemic and primary erythroid cells. Together, these findings clarify the isoform-specific regulation of HU activity by NOS2 and NOS3, revealing novel opportunities for targeted therapies of NO signaling in hematologic and vascular contexts.

## 2. Materials and Methods

### 2.1. Cell Culture

HEL92.1.7 cells (ATCC, TIB-180, Manassas, VA, USA) were grown in RPMI-1640 medium supplemented with 10% FBS, 1% glutamine, and 1% penicillin/streptomycin at 37 °C and 5% CO_2_. In the experiments, 2 × 10^6^ cells were seeded in 6-well plates and treated with HU (Abcam, ab142613, Cambridge, UK), uprosertib (UPS, Cayman Chemicals, 1-800-364-9897, Ann Arbor, MI, USA), CSD (amino acids 82-101 fused at the N-terminus to the cell-permeable Antennapedia internalization sequence 43–58, EMD Millipore, 219482-1MG, Burlington, MA, USA), or diphenyleneiodonium chloride (DPI, Sigma-Aldrich, D2926-1MG, St. Louis, MO, USA) for 5 min, 15 min, 30 min, or 48 h. Cells were collected for flow cytometry, immunocytochemistry (or processed for citrulline), nitrite measurements, and Western blot analysis. Cells were treated with 1 or 5 µM CSD [18] or with DPI at concentrations of 1, 5, and 10 µM, based on the available literature data [19], and inhibition constant (Ki). Dose–response studies were performed to ensure isoform selectivity (Supplementary Table S1). NOS3 knockdown (NOS3_kd_) HEL92.1.7 cells were generated by spinoculating 4 × 10^5^ HEL92.1.7. cells with lentiviral particles encoding NOS3-targeting short hairpin RNA (shRNA MISSION^®^, Sigma-Aldrich) at a multiplicity of infection of 1. Spinoculation was performed at 800× *g* at 37 °C for 30 min. Cells were then resuspended in a growth medium and incubated for 24 h. Transduced cells were selected with 8 µg/mL puromycin for 7 days. To achieve a population at >96% purity, green fluorescent protein (GFP)-positive NOS3_kd_ cells were sorted using a Becton Dickinson (BD) fluorescence-activated cell-sorting (FACS) Melody cell sorter (Franklin Lakes, NJ, USA).

### 2.2. Western Blot

Whole-cell lysates were prepared using RIPA buffer (50 mM Tris-HCl pH 7.5, 150 mM NaCl, 1% NP-40, 0.1% SDS, 1% sodium deoxycholate, 1 mM EDTA, and protease inhibitors). Proteins were separated on SDS-PAGE and transferred onto polyvinylidene difluoride membranes (GE Healthcare, Chicago, IL, USA). The membranes were incubated overnight at 4 °C with primary antibodies against phospho-NOS3 (Ser1177, Elabscience, E-AB-21092, Houston, TX, USA), NOS3 (Elabscience, E-AB-32268), phospho-AKT1 (Tyr564, cell signaling, 9018, Danvers, MA, USA), AKT1 (cell signaling, 2938), or β-actin (MAB8928, R&D Systems, Minneapolis, MN, USA). Horseradish peroxidase-conjugated secondary antibodies (Elabscience) were used with enhanced chemiluminescence (Bio-Rad, Hercules, CA, USA), and images were captured using a ChemiDoc Imager (Bio-Rad). Band intensities were quantified with the ImageLab software 6.0.1 (Bio-Rad).

### 2.3. Immunocytochemistry

Cells (2 × 10^5^ HEL92.1.7 or 5 × 10^5^ mouse erythroid progenitors, mERPs) were attached to slides via a cytospin and fixed in methanol at room temperature. After quenching endogenous peroxidase with 3% H_2_O_2_ in phosphate-buffered saline (PBS), the slides were incubated overnight at 4 °C in a humidified chamber with the following primary antibodies: anti-Antigen Kiel 67 (Ki67, Dako, M7187, Glostrup, Denmark), anti-single-stranded DNA (ssDNA, Abcam, ab29585), anti-NOS3 (Santa Cruz Biotechnologies, sc-654, Dallas, TX, USA), and anti-caspase 3 (Cas3, Novus Biologicals, NB100-56113, Centennial, CO, USA) antibodies. Detection was performed using the UltraVision Quato HRP detection system (TL-060-QHL; Thermo Fisher Scientific, Waltham, MA, USA), visualized with 3,3′-diaminodbenzidine substrate (Biotium, 30015, Fremont, CA, USA) and counterstained with Harris hematoxylin (HHS32-1L, Sigma-Aldrich). The samples were examined and photographed using a phase-contrast microscope (Olympus Provis AX70, Tokyo, Japan). The percentage of positive cells was quantified relative to the total number of nuclei in 10 randomly selected fields using ImageJ software 1.54c.

### 2.4. In Vitro Enzymatic Assay

The in vitro enzymatic assay was performed using an NOS activity assay kit (Cayman Chemical, 781001). The reaction mixture consisted of 25 mM Tris-HCl, pH 7.4, 3 μM tetrahydrobiopterin, 1 μM flavin adenine dinucleotide, 1 μM flavin adenine mononucleotide, 2.5 mM L-arginine, 1.25 mM nicotinamide adenine dinucleotide phosphate, 0.75 mM calcium chloride, and 1 µM calmodulin. Human recombinant NOS3 protein (OriGene, TP309228M, Rockville, MD, USA) was added to reach a final concentration of 0.5 µg/µL in the presence of 0, 10, 50, or 100 μM HU. The negative control was heat-inactivated at 95 °C for 5 min. After 10, 20, or 40 min at 37 °C, the reaction was stopped with a stop buffer (50 mM HEPES, pH 5.5, and 5 mM EDTA), and nitrite and citrulline levels were measured as described previously [17].

### 2.5. Citrulline and NO Measurement

Citrulline levels were determined using the Homocitrulline/Citrulline Assay Kit (Abcam, ab242292) with absorbance measured at 540 nm on a Rayto spectrophotometer (Shenzhen, China). NO measurement was performed using the fluorometric Nitric Oxide Synthase Activity Assay Kit (Abcam, ab211084), with fluorescence read on a VICTOR2™ D fluorometer (PerkinElmer, Waltham, MA, USA). Concentrations were calculated using the standard curve method.

### 2.6. Molecular Docking Simulation and Molecular Dynamics

The molecular docking protocol has been described in detail previously [17]. Briefly, the crystal structure of NOS3 (PDB ID: 4d1o) [20] was extracted from the RCSB Protein Data Bank in PDB format. The Lamarckian genetic algorithm method was used for protein–ligand flexible docking [21]. The grid is centered with dimensions of 15.523 × 241.24 × 22.346 Å3 in the -x, -y, and -z directions of the NOS3 protein structure, which were used to cover the protein binding sites and allow the ligands to move freely. The interactions between the target proteins and HU were analyzed and visualized as 3D results in Discovery Studio 4.0 and AutoDockTools 1.5.6 (MGLTools). The best-docked complexes, NOS3-HU, served as the initial model for molecular dynamics simulations.

### 2.7. Animal Experiments

Congenic C57BL/6J wild-type and Nos3 knockout mice (Nos3^-/-^) (Jackson Laboratory, Stock# 002684, Bar Harbor, ME, USA) were housed at the Rodent Housing Facility of the Institute for Medical Research in a temperature- and humidity-controlled environment with a 12 h light/dark cycle (to reduce confounding variables), with food and water ad libitum. Nos3^-/-^ mice are viable and fertile with elevated blood pressure [22]. A total of 85 animals (9–11-week-old male littermates) were used in the experiments. The animal group size (*n* = 3 per condition) were selected based on previous studies [17], adhering to the 3R principles of animal use reduction. HU was administered in drinking water at 1 mg/mL and replaced every 3 days for 2 weeks. On day 11 of treatment, 1 mg/kg DPI [23] or 0.5 mg/kg CSD was injected intraperitoneally twice daily for 3 days. Vehicle-treated animals were used as controls. The animals were randomly assigned to the control and treatment groups using simple random allocation. Ex vivo assays were conducted in technical triplicate to ensure reproducibility. 

### 2.8. Genotyping of Nos3^-/-^ Mice

Genomic DNA was extracted from mouse tail biopsies following phenol–chloroform isolation. The digestion buffer included 100 mM NaCl, 10 mM Tris-HCl (pH 8.0), 25 mM EDTA, 0.5% SDS, and 10 µg/µL proteinase K. PCR genotyping was performed according to the Jackson Laboratory protocols using the following primers: AAT TCG CCA ATG ACA AGA CG (*Nos3* common), AGG GGA ACA AGC CCA GTA GT (Nos3 WT), and CTT GTC CCC TAG GCA CCT CT (*Nos3* mutant). Amplicons were separated on 1.5% agarose gels stained with MidoriGreen (Nippon Genetics, Tokyo, Japan) and visualized on a ChemiDoc Imager (Bio-Rad).

### 2.9. Immunomagnetic Separation of mERPs

Bone marrow cells were resuspended in MACS buffer (1% PBS, 0.5% BSA, 2 mM EDTA) and stained with anti-human CD71-PE REAfinityTM antibody (Miltenyi Biotec, 130-120-809, Bergisch Gladbach, Germany) for 15 min at 4 °C. After washing, the cells were labeled with anti-PE MicroBeads for 15 min at 4 °C and separated using an MS column on a magnetic stand (Miltenyi Biotec). The columns were washed and separated from the magnet, and CD71-positive cells were eluted after adding Multisort Release Reagent (Miltenyi Biotec, 130-090-757) for 10 min at 4 °C. The cells were resuspended in 60 μL of buffer, 30 μL of Multisort Stop Reagent (Miltenyi Biotec, 130-090-757), and 10 μL of anti-surface protein associated with glycophorin-A (Ter119) antibody (Miltenyi Biotec, 130–049–901). By separating from the magnet and eluting, the CD71+/Ter119+ population was obtained. The purity of mERPs was confirmed by flow cytometry upon staining with anti-Ter119-FITC and CD71-PE antibodies.

### 2.10. Cell Cycle Analysis

Cells pellets were washed in PBS, fixed with ice-cold ethanol drop by drop while vortexing and incubated for 2 h at 4 °C. RNA was removed by adding 7 μL of 1 mg/mL RNase, followed by incubation for 1 h at 37 °C and overnight at 4 °C. Propidium iodide (0.5 µL of 12.5 mg/mL) was added before flow cytometric analysis on a BD FACSCalibur instrument (Franklin Lakes, NJ, USA). Data were analyzed using the NovoExpress software v1.6.2 from Agilent Technologies (Santa Clara, CA, USA).

### 2.11. Annexin V/PI Apoptotic Assay

A total of 2 × 10^6^ HEL92.1.7 cells were seeded in a 6-well plate, treated with 100 μM HU for 48 h, and then harvested. The cell pellets were washed in PBS and Annexin Binding Buffer (14 mM NaCl, 0.4 mM KCl, 75 µM MgCl2, 1 mM HEPES) and treated with 10 µg/µL RNAse A for 1 h at 37 °C. The cells were stained with anti-Annexin V-FITC (BD Pharmingen, 556419, San Diego, CA, USA) or Annexin V-APC antibody (BD Pharmingen, 550475) for 30 min at 4 °C in the dark. After washing, propidium iodide (PI) (P1304MP, ThermoFisher Scientific) or ZombieGreen (BioLegend, 423111, San Diego, CA, USA) was added, and the samples were analyzed using a BD FACSCalibur flow cytometer. Data were processed using the FlowJo v10.8.1 software (Ashland, OR, USA).

### 2.12. Biotin Switch Assay

S-nitrosylated proteins were detected using the Biotin Switch Assay Kit (ab236207, Abcam) based on a modified Jaffrey method. Briefly, free thiols in cell lysates were first blocked using a blocking reagent. Proteins were then precipitated with cold acetone and resuspended in a buffer containing reducing and biotin-labeling reagents, which cleave S-NO bonds and allow for biotinylation of the newly formed thiols. After a second acetone precipitation, the labeled proteins were resuspended in wash buffer and subjected to Western blotting. Biotinylated proteins were visualized using streptavidin-HRP, imaged with ChemiDoc Imager (Bio-Rad), and quantified using the ImageLab software (Hercules, CA, USA). Nitrosylated proteins were normalized to the total protein amount measured from the Coomassie-stained membranes.

### 2.13. Colony Formation Assay

The bone marrow cells, obtained from murine femurs, were monodispersed in DMEM (Biowest, Lakewood Ranch, FL, USA) supplemented with 5% FCS (Biowest). A total of 2 × 10^5^ or 3 × 10^5^ cells were plated in methylcellulose medium containing 3 U/mL erythropoietin (MethoCult M3334, StemCell Technologies, Vancouver, BC, Canada) or medium supplemented with 50 ng/mL stem cell factor, 10 ng/mL interleukin-3 (IL-3), and 10 ng/mL IL-6 (MethoCult GF M3434, StemCell Technologies). The cells were seeded in 35 mm culture plates and incubated at 37 °C with 5% CO_2_ and >85% humidity. Colony-forming unit erythroid (CFU-E) colonies were counted after 3 days; burst-forming unit erythroid (BFU-E) and colony–forming unit granulocyte/macrophage (CFU-GM) colonies were scored after 14 days using an inverted microscope at 4× magnification.

### 2.14. Data Analysis

All data were presented as the positive standard error of mean (SEM). Normality was assessed using the Shapiro–Wilk test and through visual inspection of the residual plots. Homogeneity of variances was evaluated using Levene’s test. If data deviated from normal distribution or equal variance assumptions, non-parametric alternatives (Mann–Whitney U test or Kruskal–Wallis test) were used, as appropriate. Significance was determined via a two-tailed Student’s *t*-test or one-way ANOVA (analysis of variance) using GraphPad Prism version 8.0.0 for Windows (GraphPad Software Inc., San Diego, CA, USA); *p*-values were set as follows: * *p* < 0.05, ** *p* < 0.01, and *** *p* < 0.001.

## 3. Results

### 3.1. Hydroxyurea Induces NOS3 Expression and Activity in HEL92.1.7 Cells

**Figure 1:**
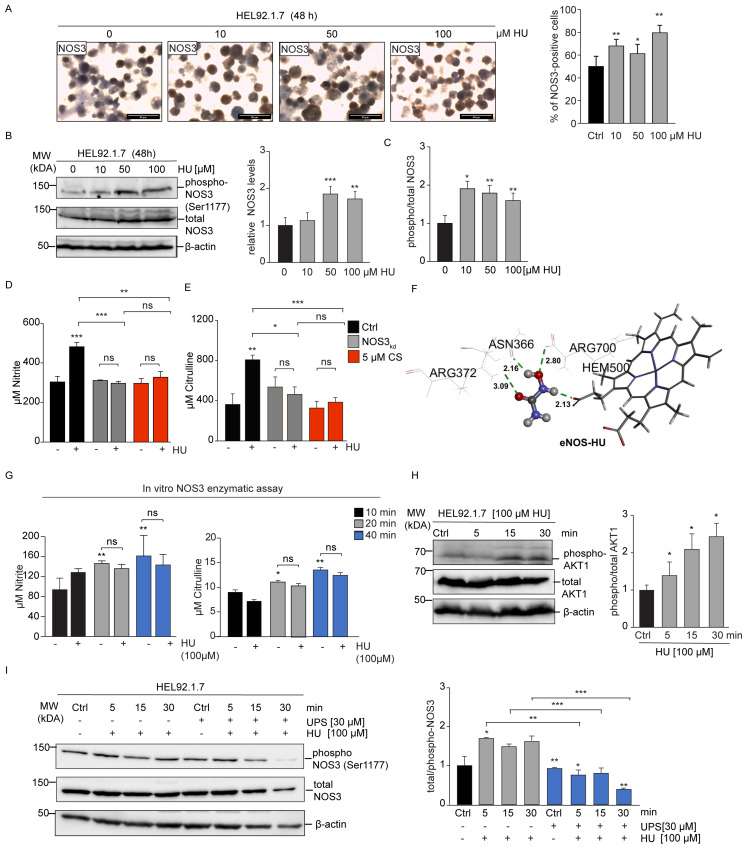
Hydroxyurea induces NOS3 expression and activity in HEL92.1.7 cells. (**A**) Immunocytochemistry for endothelial nitric oxide synthase (NOS3) protein in HEL92.1.7 cells treated with 10, 50, and 100 µM hydroxyurea (HU) or vehicle (Ctrl) for 48 h and quantification of NOS3-positive cells. Scale bar = 80 µm. (**B**) Western blot for total and phosphorylated (S1177) NOS3 protein in HEL92.1.7 cells treated with the indicated concentrations of HU or vehicle (Ctrl) for 48 h. Quantification of band intensity normalized to Ctrl, where β-actin was used as a loading control. (**C**) Quantification of phospho-to-total protein ratio normalized to Ctrl. Concentrations of (**D**) nitrite and (**E**) citrulline in HEL92.1.7 cells treated for 48 h with 5 μM of the NOS3 inhibitor Caveolin-1 scaffolding domain peptide (CSD), as well as NOS3_kd_ cells treated for 48 h with 100 µM HU or vehicle. (**F**) In silico model of HU and NOS3 interaction showing binding at amino acids ASN366 and ARG372 and with the substrate L-arginine (ARG700). In the hydroxyurea molecule, white spheres represent hydrogen, blue spheres nitrogen, red spheres oxygen, and black sphere carbon. (**G**) Concentrations of nitrite or citrulline measured after in vitro NOS3 enzymatic assay with the indicated concentrations of HU and incubation times. (**H**) Western blot for phospho-AKT1 (Ser473) and total AKT1 protein in HEL92.1.7 cells treated with 100 μM HU for 5, 15, or 30 min. Quantification of phospho-to-total protein ratio normalized to Ctrl. (**I**) Western blot for NOS3 protein in HEL92.1.7 cells treated with the indicated concentrations of HU and/or 30 μM of the AKT inhibitor uprosertib (UPS). Quantification of band intensity with β-actin used as a loading control and normalized to Ctrl. *n* = 3; mean + SEM; * *p* < 0.05, ** *p* < 0.01, and *** *p* < 0.001 vs. Ctrl or as indicated. ns—non-significant.

To assess the effect of HU on NOS3 protein level, we performed immunocytochemistry and Western blot analyses in HU-treated erythroleukemic HEL92.1.7 cells. HEL92.1.7 erythroleukemic cells represent a well-characterized, proliferative erythroid model for examining hydroxyurea-induced replication stress seen in myeloproliferative diseases. Treatment with 10 µM and 100 µM HU increased the number of NOS3-expressing cells to ~20% and ~30%, respectively, compared to the vehicle-treated control (Figure 1A), while Western blot revealed ~2-fold increase in NOS3 level with 50 and 100 µM HU (Figure 1B). These results suggest dose-dependent NOS3 regulation, where 10 µM HU induces strong upregulation in a subset of cells, while 50 µM HU promotes broad but less intense NOS3 expression in HEL92.1.7 cells. Enzymatic activity of NOS3 was assessed by measuring phosphorylated NOS3 (Ser1177). The ratio of phosphorylated to total NOS3 protein increased ~2-fold after 48 h of incubation with HU compared to the vehicle-treated control (Figure 1C). To determine the involvement of NOS3 in HU-induced NO production, we measured nitrite concentration in NOS3_kd_ and HEL92.1.7 cells treated with the NOS3 inhibitor CSD, either alone or in combination with HU. HU treatment induced NO production relative to the vehicle-treated control (481.43 ± 22.76 vs. 303.39 ± 28.41 µM, Figure 1D). NOS3_kd_ led to ~1.47-fold decrease, while CSD caused ~1.64-fold decrease in nitrite level compared to HU-treated HEL92.1.7 cells (Figure 1D). Similarly, HU increased the citrulline level relative to the control (806.67 ± 47.97 vs. 370 ± 53.69 µM), while NOS3_kd_ and CSD decreased HU-induced citrulline production by approximately 1.75-fold and 2-fold, respectively (Figure 1E).

Molecular docking simulations were performed to estimate the molecular interactions between HU and amino acids at the active site of NOS3. The binding affinity of HU to the active site of NOS3 was found to be −14.5 kJ/mol with an inhibition constant (Ki) of 2.92 mM. At the active site of NOS3, amino acids ASN366 and ARG372 formed strong hydrogen bonds with HU and the NOS3 substrate L-arginine (ARG700), with bond lengths ranging from 1.85 to 3.09 Å (Figure 1F). The structure obtained from the molecular docking simulation was used for molecular dynamics analyses, including Root Mean Square Deviation (RMSD), Root Mean Square Fluctuation (RMSF), and radius of gyration (Rg) (Supplementary Figure S1A–C) to examine overall stability, local residue, and general structure fluctuations. The average RMSD value for the NOS3-HU complex (8.61 ± 0.98) was significantly higher than for unbound NOS3 (6.89 ± 0.3), indicating a significant decrease in rigidity after HU binding to the active site (Supplementary Figure S1A). The average RMSF value for the 5746 amino acids of NOS3-HU was 0.32 ± 0.1 nm, slightly higher than for unbound NOS3 (0.29 ± 0.1 nm). In the active site of NOS3-HU, the amino acids ALA262, ASN354, and ARG700 showed intensive oscillations (Supplementary Figure S1B). The average Rg value of the NOS3-HU complex (2.42 ± 0.04 nm) was slightly higher than that of unbound NOS3 (2.4 ± 0.04 nm, Supplementary Figure S1C), indicating larger conformational changes in the secondary structure of NOS3 after HU is bound to the active site.

The NOS3-HU complex obtained from the molecular docking was further subjected to energy contribution analysis using the gmx MM/PBSA protocol based on Van der Waals, electrostatic, polar solvation, and nonpolar solvation energies [24]. The Gibbs free binding energy (ΔG_binding_) of NOS3-HU (−62.3 ± 1.89 kJ/mol) was significantly higher in absolute value than that of NOS2-HU (−24.3 ± 2.53 kJ/mol), indicating the higher binding affinity of HU towards NOS3 than NOS2 [17]. The energy of electrostatic interactions (ΔE_elec_) for NOS3-HU (−101.7 ± 1.64 kJ/mol) was much more prominent compared to NOS2-HU (−41 ± 2.27 kJ/mol) [17]. The electrostatic polar energy negatively contributed to the binding process, and it was found to be much higher for the NOS3-HU complex (84.5 ± 0.76 kJ/mol) than the NOS2-HU complex (54.8 ± 0.92 kJ/mol). The value of favorable non-electrostatic nonpolar free energy (ΔG_nonpolar_) for the NOS3-HU complex (146.9 ± 1.77 kJ/mol) was double that for the NOS2-HU complex (−79.1 ± 2.36 kJ/mol), significantly contributing to total binding free energy [17]. A negative value of −45.2 ± 0.22 kJ/mol indicates an attractive and stable interaction between NOS3 and HU, which is still higher compared to that of the NOS2-HU complex (−38.1 ± 0.02 kJ/mol) [17]. Based on the obtained results, the ΔE_elec_ and ΔG_nonpolar_ were the major contributors to the total binding free energy and led to the difference in the binding affinity of HU to NOS3 and NOS2.

To determine whether HU directly regulates NOS3 activity, we conducted an in vitro enzymatic assay using human recombinant NOS3 protein, the substrate L-arginine, the required cofactors, and increasing concentrations of HU (10, 50, and 100 µM) or vehicle control, and we measured the concentration of NO and citrulline. The concentrations of nitrite and citrulline did not significantly change in response to HU treatment with increasing incubation period (Figure 1G) or increasing HU concentrations (Supplementary Figure S1D). Although the levels of both reaction products increased with longer incubation times, HU treatment did not significantly alter their levels (Figure 1G). Given these findings, we investigated whether HU may modulate NOS3 activity indirectly via AKT1, a kinase known to phosphorylate NOS3 [25]. HU treatment led to a time-dependent increase in the phospho-AKT1/total AKT1 ratio (Figure 1H). Furthermore, the inhibition of AKT1 with UPS reduced HU-induced NOS3 phosphorylation (Figure 1I), supporting a role for AKT1 in mediating HU-dependent NOS3 activation.

### 3.2. NOS3 Depletion or Inhibition Shifts Cells from S to G_0_/G_1_ Phase and Regulates Apoptosis Under HU Treatment

**Figure 2:**
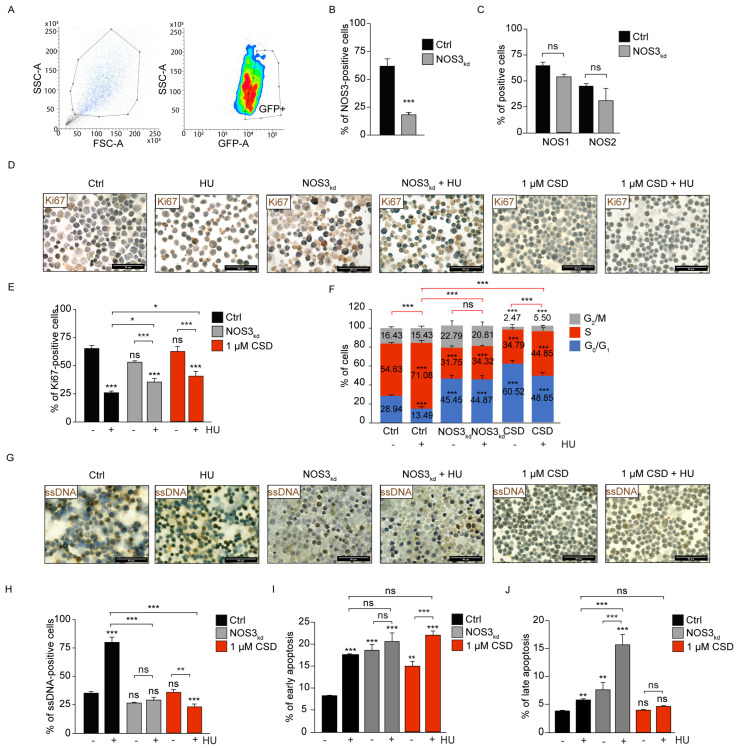
NOS3 deletion or inhibition shifts cells from S to G_0_/G_1_ phase and regulates apoptosis under hydroxyurea treatment. (**A**) Sorting of GFP-positive endothelial nitric oxide synthase knock-down (NOS3_kd_) HEL92.1.7 cells after transduction with lentiviral particles containing shRNA against NOS3 and GFP. (**B**) NOS3_kd_ was confirmed by quantifying NOS3-positive cells upon immunocytochemistry staining. (**C**) Quantification of NOS1- and NOS2-positive cells in NOS3_kd_ and control HEL92.1.7 cells after immunocytochemistry staining. HEL92.1.7 and NOS3_kd_ HEL92.1.7 cells were treated with 100 μM hydroxyurea (HU), 1 μM of the NOS3 inhibitor Caveolin-1 scaffolding domain peptide (CSD), or vehicle. (**D**) Immunocytochemistry for Ki67 protein and (**E**) quantification of Ki67-positive cells. (**F**) Percentage of cells in the G_0_/G_1_, S, and G_2_/M phases of the cell cycle were analyzed by flow cytometry after PI staining. (**G**) Immunocytochemistry for ssDNA and (**H**) quantification of ssDNA-positive cells; percentage of (**I**) early and (**J**) late apoptotic cells were analyzed by flow cytometry after Annexin V/PI staining. (**B**–**E**,**G**,**H**) *n* = 5; (**E**,**I**,**J**) *n* = 3; mean + SEM; * *p* < 0.05, ** *p* < 0.01, and *** *p* < 0.001 vs. control (Ctrl) or as indicated (red line refers to S phase). ns—non-significant. Scale bar = 80 µm.

A stable NOS3_kd_ HEL92.1.7 clone was generated using shRNA directed against NOS3, followed by FACS sorting for GFP-positive transfected cells (Figure 2A). The percentage of NOS3-expressing cells was strongly reduced in NOS3_kd_ compared to the control (64.18 ± 5.11 vs. 20.09 ± 2.48%; Figure 2B). Notably, NOS3_kd_ did not affect neuronal NOS (NOS1) or NOS2 levels (Figure 2C). To investigate the role of NOS3 in HU-mediated proliferation, NOS3_kd_ cells were treated with HU, and HEL92.1.7 cells were treated with CSD only or in combination with HU. This was followed by immunocytochemistry to detect the proliferation marker Ki67 (a nuclear protein expressed during all active phases of the cell cycle) and cell cycle analysis via flow cytometry. HU treatment decreased the number of Ki67-expressing HEL92.1.7 cells compared to the untreated control (25.88 ± 1.23 vs. 61.02 ± 0.15%; Figure 2D,E). Treatment with NOS3_kd_ and CSD partially rescued the HU-induced suppression of cell proliferation compared to HU treatment (32.91 ± 2.49 and 40.61 ± 3.05 vs. 25.88 ± 1.23%, respectively; Figure 2D,E). Compared to untreated cells, HU treatment combined with NOS3_kd_ decreased the number of Ki67-expressing cells (28.98 ± 2.46 vs. 53.04 ± 1.25%). Similarly, combined HU and CSD treatment decreased cell proliferation compared to the treatment with CSD alone (40.61 ± 3.05 vs. 60.25 ± 1.89%; Figure 2D,E). HU induced replication stress, leading to the accumulation of cells in the S-phase of the cycle compared to vehicle-treated control (54.63 ± 1.15 vs. 71.08 ± 2.84%). HU-treated NOS3_kd_ cells showed a reduced S-phase fraction (34.32 ± 0.73 vs. 71.08 ± 2.84%) and an increased G_0_/G_1-_phase fraction (44.87 ± 3.75 vs. 13.49 ± 1.79%) compared to HU-treated control cells (Figure 2F and Figure S2A). NOS3 inhibition by CSD similarly impaired HU-induced S-phase arrest (44.85 ± 2.13 vs. 71.08 ± 2.84%), leading to the increased number of cells in the G_0_/G_1_-phase of the cell cycle (48.85 ± 1.56 vs. 13.49 ± 1.79%; Figure 2F and Figure S2A). Together, these results suggest that HU inhibition of Ki67 expression was partially rescued by NOS3 silencing and inhibition with a parallel S-G_0_/G_1_ shift.

We further examined the effect of NOS3kd and CSD inhibition on HU-induced DNA damage and apoptosis in HEL92.1.7 cells. For this purpose, we analyzed the presence of ssDNA, a marker of replication stress, DNA damage, and apoptosis, via immunocytochemistry or performed Annexin V/PI apoptotic assay via flow cytometry. HU treatment led to a strong increase in ssDNA level compared to the control (79.56 ± 3.19 vs. 32.56 ± 2.52%, Figure 2G,H). NOS3kd and CSD strongly decreased the number of ssDNA-positive cells in the presence of HU compared to HU treatment alone (28.22 ± 2.57 and 24.83 ± 2.05 vs. 79.56 ± 3.19%, respectively, Figure 2G,H). HU treatment did not affect the ssDNA level in NOS3kd cells (28.22 ± 2.57 vs. 25.34 ± 1.51%) but further reduced it in CSD-treated cells (24.83 ± 2.05 vs. 31.65 ± 1.84%; Figure 2G,H) compared to NOS3kd or CSD alone. Moreover, the number of HU-treated NOS3kd early apoptotic cells was not significantly changed compared to HU treatment alone (20.6 ± 0.88 vs. 17.38 ± 2.01%; Figure 2I and Figure S2B) in the Annexin V/PI assay. In addition, the percentage of HU-treated NOS3kd cells in late apoptosis was significantly increased compared to HU-treated cells with endogenous NOS3 levels (16.53 ± 1.95 vs. 6.19 ± 0.21%; Figure 2J), indicating increased total apoptosis (37.13 ± 1.09 vs. 23.57 ± 2.09%; Supplementary Figure S2B,C). The combined CSD and HU treatment did not affect early (21.74 ± 1.07 vs. 17.38 ± 2.01%; Figure 2I) nor late apoptosis compared to HU treatment (4.83 ± 0.43 vs. 6.19 ± 0.21%; Figure 2J), evidenced by an unchanged percentage of total apoptotic cells (26.57 ± 1.31 vs. 23.57 ± 2.09%; Supplementary Figure S2B,C). Both NOS3 depletion and inhibition rescued HU-induced replication stress/DNA damage as determined by ssDNA levels, while only NOS3_kd_ increased late apoptosis, as determined by Annexin V/PI assay. To quantitatively assess the balance between proliferation and apoptosis under HU stress, we compared the relative fractions of actively proliferating Ki67-positive cells and Annexin V^+^/PI± total apoptotic cells by calculating the proliferation-to-apoptosis index (PAI) in control, NOS3kd, and CSD-treated HEL92.1.7 cells in the presence or absence of HU. The HU-treated control and NOS3kd HEL92.1.7 cells exhibited PAI < 1, indicating cytotoxicity, while CSD-treated cells showed a proliferative outcome, with PAI > 1 (Supplementary Figure S2D). In summary, NOS3 modulation reduced both ssDNA accumulation and the HU-induced S-phase arrest, suggesting that NOS3 mainly contributes to replication stress under HU exposure (Supplementary Table S2).

### 3.3. NOS3 Deficiency Impairs HU-Induced Protein Nitrosylation and Alters Hematopoietic Lineage Commitment In Vivo

**Figure 3:**
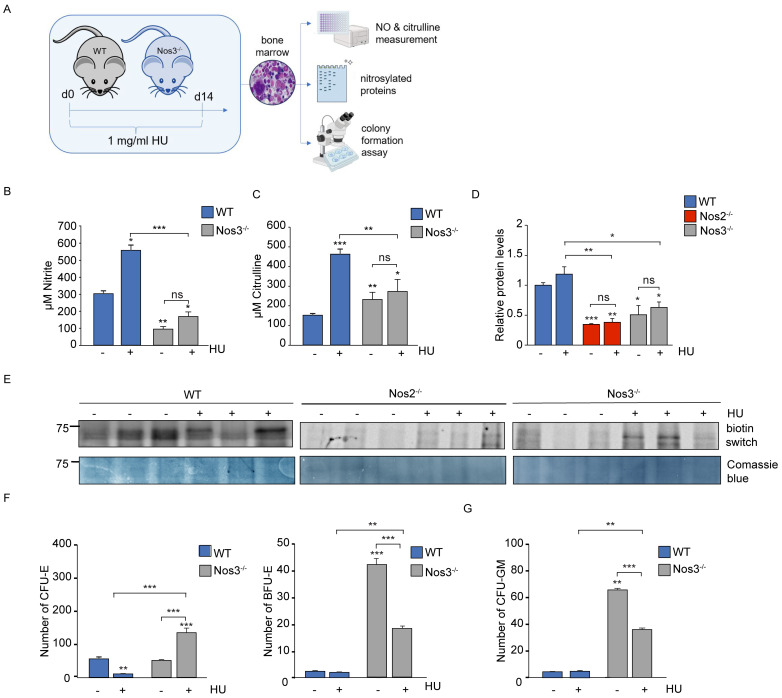
Nos3 deficiency impairs hydroxyurea-induced protein nitrosylation and alters hematopoietic lineage commitment in vivo. (**A**) Schematic representation of experimental setup: endothelial nitric oxide synthase null mice (Nos3)^-/-^ or wild-type (WT) mice were treated orally with 1 mg/mL hydroxyurea (HU) or vehicle in drinking water for 2 weeks. Bone marrow cells were used for NO and citrulline measurements, biotin switch assay for the detection of nitrosylated proteins, and colony formation assay. Concentrations of (**B**) nitrite and (**C**) citrulline in the bone marrow of WT and Nos3^-/-^ mice treated with HU or vehicle. (**D**) Quantification of nitrosylated proteins in bone marrow of WT, Nos2^-/-^, and Nos3^-/-^ mice treated with HU or vehicle. Total protein was used as a loading control, and protein levels were normalized to the levels of WT mice. (**E**) Nitrosylation of proteins was visualized using anti-streptavidin-HRP antibody after the biotin switch assay, while Coomassie blue staining was used to detect total proteins. (**F**) Colony formation assay showing the number of late erythroid (CFU-E), early erythroid (BFU-E), and (**G**) granulocyte/macrophage progenitors (CFU-GM) in the bone marrow of WT and Nos3^-/-^ mice treated with vehicle and HU, respectively, for 2 weeks. *n* = 3; mean + SEM; * *p* < 0.05, ** *p* < 0.01, and *** *p* < 0.001 vs. untreated WT or as indicated. ns—non-significant.

To elucidate the role of the Nos3 protein in bone marrow during in vivo HU treatment, Nos3 null mice or their wild-type (WT) littermates were given 1 mg/mL HU orally for 14 days (Figure 3A). Homozygous disruption of the *Nos3* gene was confirmed by genotyping (Supplementary Figure S3A), and depletion of Nos3 protein in bone marrow was confirmed by immunocytochemistry (Supplementary Figure S3B). HU treatment increased the levels of nitrite (536.8 ± 36.77 vs. 315.49 ± 33.39 µM; Figure 3B) and citrulline (471 ± 45.37 vs. 199.5 ± 45.37 µM; Figure 3C) in the bone marrow of the WT mice compared to the vehicle-treated mice. Nos3 deficiency with HU treatment decreased the levels of nitrite (3.61-fold; Figure 3B) and citrulline (1.85-fold; Figure 3C) compared to HU treatment alone. Nitrite and citrulline levels did not significantly differ between HU-treated and untreated Nos3^-/-^ mice (Figure 3B,C).

Since HU increases NO levels by acting as a NO donor and stimulating NOS enzymes, we next investigated if HU alters protein nitrosylation in the bone marrow of mice using the biotin switch assay (Figure 3D,E). Proteins at ~60 kDa were highly nitrosylated in the bone marrow of HU-treated WT mice but were hardly detectable in the bone marrow of Nos2^-/-^ and Nos3^-/-^ mice, with a slight increase observed upon HU treatment (Figure 3D,E), indicating that both NOS isoforms are involved in HU-mediated nitrosylation. For another band of proteins at ~40 kDa, nitrosylation was decreased by Nos2^-/-^ (*p* < 0.05) and not by Nos3^-/-^, while HU treatment led to an increase in nitrosylation independent of Nos2/Nos3 (Supplementary Figure S3C,D). These results show that HU induces protein nitrosylation in the bone marrow via both Nos2-/Nos3-dependent and -independent pathways.

To examine the effect of Nos3 deficiency on hematopoiesis in HU-treated mice, we performed a colony formation assay. HU treatment led to a decrease in late erythroid progenitors (CFU-E) compared to the level observed in untreated mice, but this reduction was rescued in HU-treated Nos3^-/-^ mice (Figure 3F). Moreover, Nos3^-/-^ led to a robust increase in early erythroid progenitors (BFU-E) compared to the level seen in WT controls (Figure 3F), indicating impaired erythropoiesis, along with favored myeloid lineage differentiation, as shown by the increased number of granulocyte/macrophage progenitors (CFU-GM, Figure 3G). Treatment of Nos3^-/-^ mice with HU decreased the numbers of BFU-E and CFU-GM compared to those seen in untreated Nos3^-/-^ mice, but remained significantly higher than in the HU-treated WT controls. In contrast to CFU-E, Nos3 deficiency elevated BFU-E and CFU-GM and did not prevent their reduction by HU (Figure 3F,G).

### 3.4. In Vivo NOS3 Depletion or Inhibition Impairs HU-Mediated S-Phase Arrest and Alters Apoptosis

**Figure 4:**
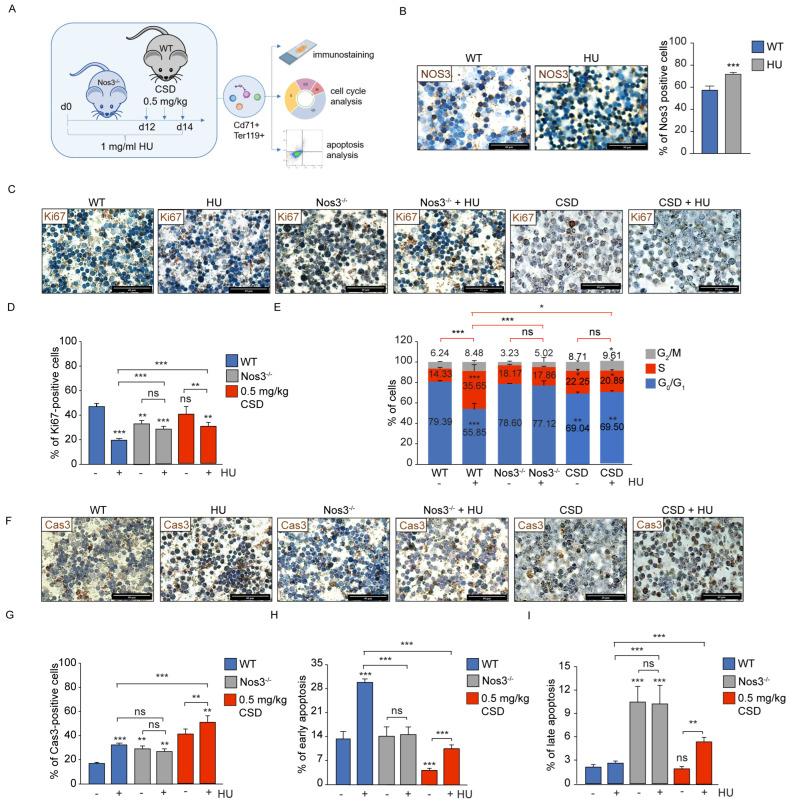
In vivo NOS3 depletion or inhibition impairs hydroxyurea-mediated S-phase blockage and alters apoptosis. (**A**) Schematic representation of experimental setup: endothelial nitric oxide synthase (Nos3)^-/-^ and wild-type (WT) mice were treated orally with 1 mg/mL hydroxyurea (HU) and vehicle, respectively, in drinking water for 2 weeks. WT mice were treated with 0.5 mg/kg CSD intraperitoneally on days 12–14 of HU treatment. Mouse erythroid progenitors (mERPs) isolated from WT and Nos3^-/-^ mice based on CD71 and Ter119 expression and used for immunostaining, and cell cycle and apoptosis analysis. (**B**) Immunocytochemistry for endothelial nitric oxide synthase (NOS3) protein and quantification of NOS3-positive cells. (**C**) Immunocytochemistry for Ki67 and (**D**) quantification of Ki67-positive cells. (**E**) Cell cycle distribution by flow cytometry showing percentages of cells in the G_0_/G_1_, S, and G_2_/M phases. (**F**) Immunocytochemistry for caspase 3 (Cas3) and (**G**) quantification of Cas3-positive cells. Annexin V/PI assay showing percentages of: (**H**) early and (**I**) late apoptotic cells. n = 3; mean + SEM; * *p* < 0.05, ** *p* < 0.01, and *** *p* < 0.001 vs. untreated WT or as indicated (red line refers to S phase). ns—non-significant. Scale bar = 40 µm.

To elucidate the role of Nos3 protein in HU-mediated cytostasis of erythroid cells, we treated Nos3^-/-^ and WT mice with HU or vehicle or WT mice with the Nos3 inhibitor CSD; we then performed immunomagnetic isolation of mERPs from the bone marrow and analyses of proliferation, cell cycle, and apoptosis (Figure 4A). The purity of the isolated cell population was verified via flow cytometry based on the expression of CD71 and Ter119 erythroid markers (Supplementary Figure S4A). HU treatment induced a ~20% increase in NOS3-expressing mERPs compared to that in untreated littermates (Figure 4B). As expected, HU treatment decreased the number of Ki67-positive mERPs compared to that of untreated WT mice (49.19 ± 1.52 vs. 19.79 ± 1.12%; Figure 4C). Nos3^-/-^ and CSD inhibition in the presence of HU partially rescued the HU-induced decrease in Ki67 level (28.43 ± 1.09 and 33.40 ± 4.39 vs. 19.79 ± 1.12%), although the level remained significantly lower compared to that of WT mice (49.19 ± 1.52%; Figure 4C,D). Moreover, HU treatment reduced the Ki67 level in CSD-treated mERPs compared to treatment with the inhibitor alone, but not the Ki67 level in Nos3-deficient mERPs (Figure 4C,D). HU-treated Nos3^-/-^ and CSD inhibition decreased the percentage of cells in the S-phase of the cell cycle compared to that seen in HU-treated WT mERPs (17.86 ± 0.89 and 20.89 ± 0.84 vs. 35.67 ± 4.25%) while increasing the percentage of cells in the G_0_/G_1-_phase (55.85 ± 5.34 vs. 77.12 ± 4.41%, and 69.50 ± 1.38%; Figure 4E and Supplementary Figure S4B). Nos3-deficiency or CSD alone had a similar effect on S and G_0_/G_1_-phase distribution as that seen in HU-treated WT mERP (18.17 ± 1.79% and 22.25 ± 1.42 vs. 35.67 ± 4.25%; Figure 4E and Figure S4B). Importantly, the cell cycle distribution of HU-treated Nos3^-/-^ mERP cells closely resembled that of untreated WT cells, indicating a diminished cytostatic response to HU in the absence of Nos3, which was also evident but not as pronounced after Nos3 inhibition. The partial rescue of Ki67 expression and the impaired S-phase arrest observed in our mouse model are consistent with the findings in HEL92.1.7 cells, confirming that Nos3 functionally impacts cell proliferation in vivo.

In WT mERPs, HU increased the quantity of cells expressing cleaved Cas3, a marker of the execution phase of apoptosis, compared to untreated mERPs (19.27 ± 1.59 vs. 29.82 ± 0.69%; Figure 4F,G). Nos3^-/-^ mERPs treated with HU showed a similar percentage of Cas3-expressing cells as in HU-treated WT mERPs (28.08 ± 0.9 vs. 29.82 ± 0.69%), while CSD inhibition increased the Cas3-positive population (49.76 ± 8.06%; Figure 4F,G). Using Annexin V/PI assay, we detected a decreased percentage of early apoptotic cells in HU-treated Nos3^-/-^ compared to HU-treated WT mERPs (14.57 ± 2.22 vs. 30.17 ± 3.47%; Figure 4H and Figure S4C), while the percentage of late apoptotic cells was increased (10.22 ± 2.39 vs. 2.68 ± 0.3%; Figure 4I and Figure S4C), so the overall effect on apoptosis was non-significant (24.78 ± 4.43 vs. 32.85 ± 3.67%; Supplementary Figure S4D). With combined HU and CSD treatment, early apoptosis was also decreased compared to HU-treated WT mERPs (9.72 ± 0.25 vs. 30.17 ± 3.47%; Figure 4H), while late apoptosis was increased (4.93 ± 0.19 vs. 2.68 ± 0.3%; Figure 4I), but to a lesser extent than in Nos3^-/-^, leading to decreased total apoptosis (14.65 ± 0.17 vs. 2.68 ± 0.3%; Supplementary Figure S4C). HU treatment did not affect early, late, or total apoptosis in Nos3-deficient mERPs relative to untreated Nos3-deficient mERPs, while combined HU and CSD treatment increased apoptosis compared to CSD treatment alone (Figure 4H,I and Figure S4C,D). Genetic deletion of Nos3 and Nos3 inhibition altered mERP apoptotic dynamics: the fraction of early apoptotic cells decreased, while cleaved Cas3 levels and late apoptotic events increased. The overall outcome of Nos3 loss/inhibition under HU stress is a net shift toward increased proliferation relative to cell death, as demonstrated by PAI ≥ 1 (Supplementary Figure S4E). These results corroborate the findings in NOS3_kd_ HEL92.1.7 cells and underline the role of Nos3 in regulating the survival of erythroid cells under HU treatment.

### 3.5. Dual NOS2/NOS3 Inhibition Impairs HU-Induced Proliferation Block in Erythroid Cells

**Figure 5:**
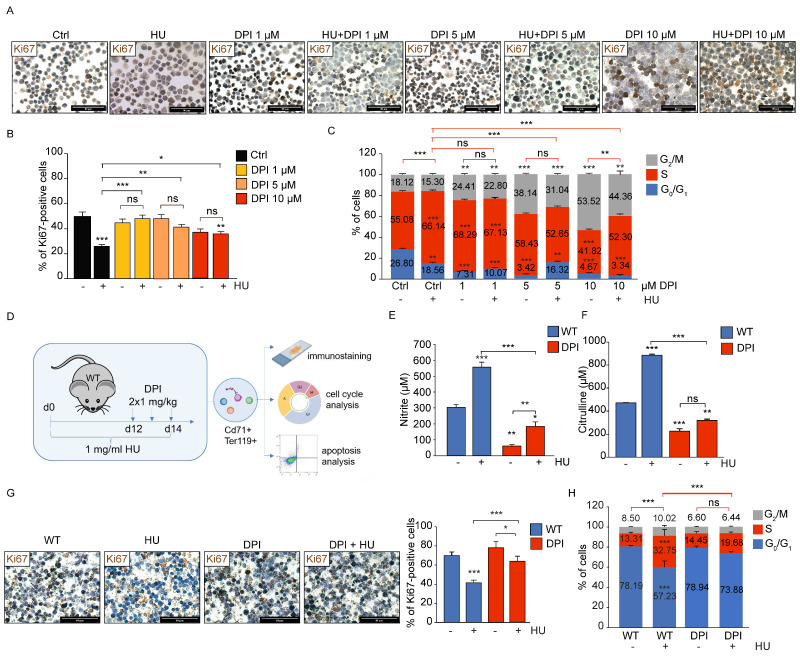
Dual NOS2/NOS3 inhibition impairs hydroxyurea-induced proliferation block in erythroid cells. HEL92.1.7 cells were treated for 48 h with the indicated concentrations of diphenyleneiodonium chloride (DPI), an NADPH oxidase (NOX)/inducible nitric oxide synthase (NOS2)/endothelial nitric oxide synthase (NOS3) inhibitor alone or in combination with 100 µM of hydroxyurea (HU). (**A**) Immunocytochemistry for Ki67 and (**B**) quantification of Ki67-positive cells were performed. Scale bar = 80 µm. (**C**) Percentages of HEL92.1.7 cells in G_0_/G_1_, S, and G_2_/M cell cycle phases were analyzed by flow cytometry after PI staining. (**D**) Schematic representation of experimental setup: wild-type (WT) mice were treated orally with 1 mg/mL HU or vehicle in drinking water for 14 days. On days 12, 13, and 14, the mice were injected with 1 mg/kg DPI twice daily. Mouse erythroid progenitors (mERPs) were isolated from WT and Nos3^-/-^ mice based on CD71 and Ter119 expression and used for nitrite and citrulline measurements, immunostaining, and apoptosis analysis. Concentrations of (**E**) nitrite and (**F**) citrulline in the bone marrow of WT mice treated with HU, DPI, or a combination of both. mERPs were isolated from WT mice treated with DPI or vehicle and (**G**) immunocytochemistry for Ki67 and quantification of Ki67-positive cells were performed. Scale bar = 40 µm. (**H**) Percentages of mERPs in G_0_/G_1_, S, and G_2_/M phases of cell cycle were analyzed by flow cytometry after staining with PI. n = 3; mean + SEM; * *p* < 0.05, ** *p* < 0.01, and *** *p* < 0.001 vs. WT or as indicated (red line refers to S phase). ns—non-significant.

NOS2 inhibition rescued HU-reduced Ki67 expression [17], whereas NOS3 inhibition slightly increased Ki67 expression and alleviated HU-induced S-phase arrest. We therefore hypothesized that simultaneous inhibition of both isoforms might exert an additive effect or even completely abolish the antiproliferative action of HU. We treated HEL92.1.7 cells with NOS2/NOS3 and the NADPH oxidase (NOX) inhibitor diphenyleneiodonium (DPI) and analyzed Ki67 expression and cell cycle distribution. Indeed, NOX/NOS inhibition with 1–10 µM DPI was sufficient to effectively rescue the HU-induced decrease in Ki67 expression (Figure 5A,B). At a low concentration of 1 µM, DPI combined with HU did not reduce the proportion of S-phase cells compared to HU alone; however, it decreased the G_0_/G_1_ population and increased the G_2_/M population (Figure 5C and Figure S5A). However, at higher concentrations, combined HU and DPI treatment significantly decreased the number of cells in the S-phase (52.65 ± 0.06 and 52.30 ± 1.83 vs. 66.14 ± 2.51%) and increased the number of cells in the G_2_/M phase (31.06 ± 0.2 and 44.36 ± 1.27 vs. 15.3 ± 2.32%) of the cell cycle compared to HU treatment alone (Figure 5C and Figure S5A). Compared to DPI-treated cells, combined DPI and HU treatment did not change the number of cells in the S-phase (Figure 5C and Figure S5A). In HEL92.1.7 cells, NOX/NOS inhibition increased the population of actively proliferating Ki67-positive cells and impaired HU-induced S-phase arrest with concomitant increase in cells in the G_2_/M phase of the cell cycle.

To test the effect of NOS2/NOS3 on HU-induced proliferation inhibition in vivo, WT mice were treated with HU orally for 14 consecutive days, and in the last 3 days, they were injected intraperitoneally with 1 mg/kg of the NOX/NOS inhibitor DPI or vehicle twice every 12 h; nitrite and citrulline measurements in the bone marrow, Ki67 immunostaining, and cell cycle analysis of mERPs were performed (Figure 5D). Oral HU treatment increased the nitrite and citrulline levels by 1.7-fold and 1.57-fold, respectively, compared to the vehicle, while combined HU and DPI treatment reduced the levels by 2.91-fold and 1.47-fold, respectively, compared to HU alone (Figure 5E,F). HU slightly increased the nitrite level, but not citrulline level in DPI-treated WT mice when compared to DPI alone (Figure 5E,F). Combined DPI and HU treatment completely rescued the number of Ki67-positive mERPs decreased by HU (Figure 5G) and decreased the percentage of mERPs in the S-phase of the cell cycle (19.68 ± 1.4 vs. 32.75 ± 1.35%) while increasing those in the G_0_/G_1_-phase (73.88 ± 1.6 vs. 57.23 ± 3.7%) compared to HU treatment alone (Figure 5H and Figure S5B). Moreover, the distribution of the cell cycle phases in DPI-/HU-treated mERPs mirrored that of untreated WT mice (Figure 5H and Figure S5B). In addition, HU decreased Ki67-positive mERPs, but did not block cells in the S-phase in DPI-treated WT mice compared to mice treated with DPI alone (Figure 5G,H). In mERP cells, Nox/Nos inhibition increased the Ki67-positive population and impaired HU-induced S-phase accumulation by increasing the G_0_/G_1_ fraction, restoring cell proliferation and cell cycle distribution to be close to the baseline. Taken together, DPI increased the cycling pool and impaired HU-induced S-phase arrest in both HEL92.1.7 and mERP cells, but differentially affected the cell cycle, increasing G_2_/M in HEL92.1.7 cells and G_0_/G_1_ in mERP cells.

### 3.6. Dual NOS2/NOS3 Inhibition Impairs HU-Induced Apoptosis of Erythroid Cells in a Context-Dependent Manner

**Figure 6:**
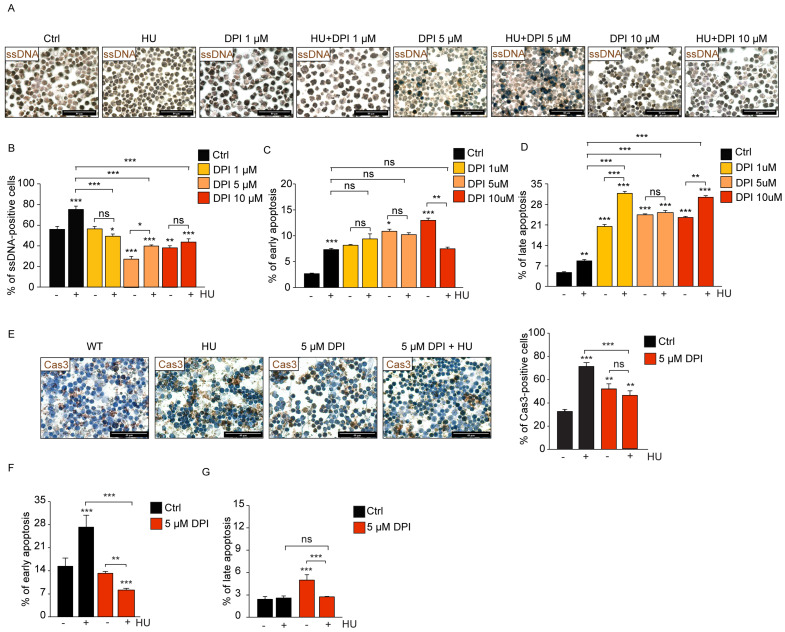
Dual NOS2/NOS3 inhibition impairs hydroxyurea-induced apoptosis of erythroid cells in a context-dependent manner. HEL92.1.7 cells were treated for 48 h with the indicated concentrations of diphenyleneiodonium chloride (DPI), an NADPH oxidase (NOX)/inducible nitric oxide synthase (NOS2)/endothelial nitric oxide synthase (NOS3) inhibitor alone or in combination with 100 µM hydroxyurea (HU). (**A**) Immunocytochemistry for ssDNA and (**B**) quantification of ssDNA-positive cells were performed. Scale bar = 80 µm. Annexin V/PI assay showing percentages of (**C**) early and (**D**) late apoptotic cells. Mouse erythroid progenitors (mERPs) isolated from WT mice treated with 1 mg/mL HU, 1 mg/kg DPI, or a combination of both were used for: (**E**) immunocytochemistry for caspase-3 (Cas3) and quantification of Cas3-positive cells. Scale bar = 40 µm. Annexin V/PI assay showing percentages of (**F**) early and (**G**) late apoptotic mERPs. n = 3; mean + SEM; * *p* < 0.05, ** *p* < 0.01, and *** *p* < 0.001 vs. WT or as indicated. ns—non-significant.

Next, we analyzed the effect of NOX/NOS inhibition on HU-induced replication stress, DNA damage, and apoptosis in HEL92.1.7 cells. Co-treatment of HEL92.1.7 cells with HU and 1–10 µM DPI led to a dose-dependent decrease in the number of ssDNA-positive cells compared to HU alone (Figure 6A,B). Annexin V/PI assay showed that DPI in combination with HU did not significantly change the percentage of early apoptotic cells (Figure 6C and Figure S6A), while the percentage of late apoptotic cells was additionally increased compared to HU alone (Figure 6D and Figure S6A). In HEL92.1.7 cells, the overall effect of combined DPI treatment and HU treatment was increased apoptosis relative to HU treatment alone (Supplementary Figure S6A,B). Combined DPI and HU treatment demonstrated dose-dependent stimulation of late apoptosis and reduction in early apoptosis when compared to DPI-treated HEL92.1.7 cells (Figure 6C,D). Overall, results obtained with DPI mirror those obtained with NOS3_kd_ HEL92.1.7 cells, including decreased replication stress and increased late apoptosis (Supplementary Table S2). In vivo DPI treatment decreased the number of HU-induced Cas3-positive mERPs compared to HU treatment alone (*p* < 0.001), but the level remained slightly higher than that for WT mERPs (Figure 6E). Annexin V/PI assay showed that DPI and HU mutually decreased early apoptotic mERPs (8.19 ± 0.51 vs. 27.41 ± 3.66%; Figure 6F), with unaltered late apoptosis (2.75 ± 0.06 vs. 2.64 ± 0.3%; Figure 6G and Figure S6C), resulting in overall decreased apoptosis (30.04 ± 3.87 vs. 10.94 ± 0.48%; Supplementary Figure S6C,D) compared to HU-treated mERPs. Combined DPI and HU treatment significantly reduced early, late, and total apoptosis of mERPs relative to that seen in DPI-treated WT mice (Figure 6F,G and Figure S6D). In HEL92.1.7 cells, DPI decreased HU-induced replication stress and DNA damage while increasing late apoptosis, whereas in mERP cells, DPI slightly reduced Cas3 expression and lowered apoptosis in the presence of HU.

## 4. Discussion

**Scheme 1:**
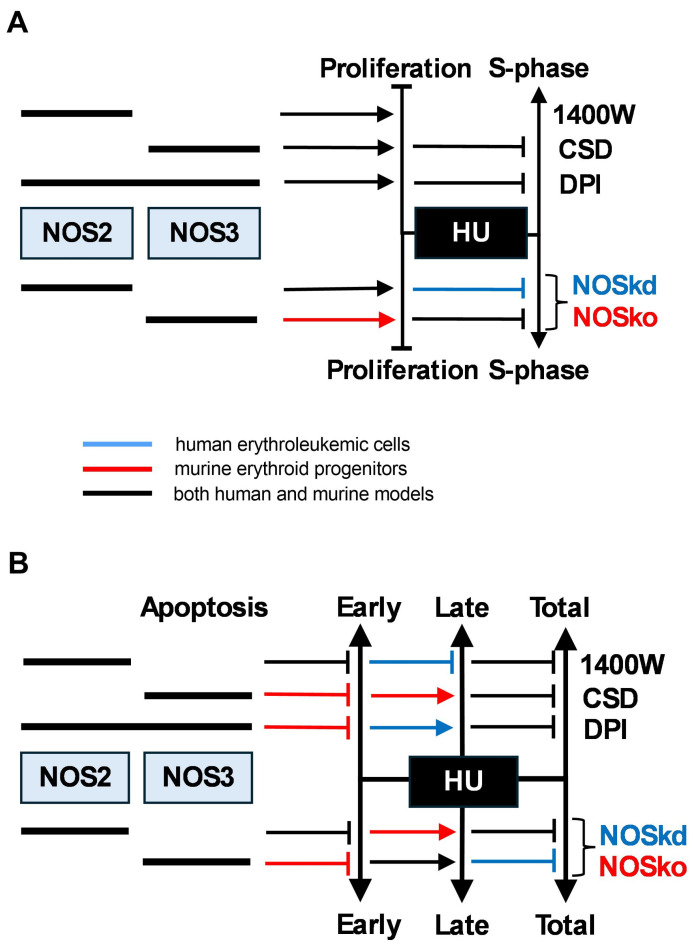
Nitric oxide synthases (NOSs) mediate hydroxyurea (HU)-induced reduction in cell proliferation and enhancement of apoptosis in erythroid cells. Compared to individual HU treatment, NOS2 and NOS3 mediate the HU effects on (**A**) cell proliferation (confirmed by the level of the Ki67 marker during active cell cycle: G_1_, S, G_2_, and mitosis) and S-phase arrest (flow cytometry); (**B**) early (flow cytometry Annexin V^+^/propidium iodide (PI)^-^ population), late (flow cytometry—Annexin V^+^/PI^+^ population), and total (ssDNA for HEL92.1.7 cells and caspase 3 for mice) apoptosis. The ssDNA accumulation may indicate DNA damage or replication stress, whereas apoptosis is more reliably supported by markers such as Caspase-3 activation and Annexin V positivity. Red line—studies on mouse erythroid progenitors (wild-type mice treated with NOS inhibitors and Nos knockout (NOS_ko_) mice); blue line—studies on HEL92.1.7 erythroleukemic cells (NOS inhibitors and NOS knockdown (NOS_kd_)); black line—studies on both models. Lines with an arrow (stimulation) and inhibition arc (reduction) represent the effects of NOS inhibitors and genetic modifications. The full lines below and above the NOS boxes correspond to the NOS2 and/or NOS3 isoforms.

HU inhibits cell proliferation and causes S-phase cell cycle arrest in a NOS-dependent manner in erythroid cells (Scheme 1A). NOS3 is essential for HU-induced S-phase arrest, while NOS2 is critical for HU-mediated inhibition of cell proliferation at the genetic and enzymatic levels in erythroid cells [17]. Combined NOS2/NOS3 activity appears to be necessary for the HU-induced inhibition of cell proliferation and S-phase arrest at the enzymatic level, emphasizing the complementary nature of these isoforms in mediating cellular outcomes. HU exhibited NOS3 and NOS2 dependence in the stimulation of early apoptosis and combined NOS2/NOS3 dependence in both early and total apoptosis of erythroid cells [17] (Scheme 1B). HU stimulation of late apoptosis was NOS3-mediated in both HEL92.1.7 and mERP cells, while its stimulation of total apoptosis was NOS3-mediated in erythroleukemic cells (Scheme 1B). HU also demonstrated NOS2 [17] and NOS3 dependence in inhibiting cell proliferation and S-phase arrest, respectively, at the genetic and enzymatic levels, as well as in stimulating apoptosis, as confirmed by the mutual NOS2/NOS3 dependence at the enzymatic level. These insights may inform future strategies to enhance HU efficacy or reduce resistance by targeting specific NOS pathways in hematologic malignancies.

To further clarify isoform-specific contributions of NOS enzymes, we compared the effects of the NOS3 inhibitor CSD with NOS2-selective inhibitor 1400 W and the pan-NOS inhibitor L-NAME, as published previously [17] (Supplementary Table S2). All three inhibitors showed comparable effects in rescuing Ki67 expression and ssDNA accumulation decreased by HU, indicating a common impact on proliferation and replication stress; however, their effects on cell cycle distribution and apoptosis differed. These differences highlight the distinct and, in part, opposing roles of the NOS2 and NOS3 isoforms in regulating cellular responses to HU. Our data indicate that NOS2 promotes apoptotic signaling, while NOS3 has a protective effect. Consequently, pan-NOS inhibition results in a complex phenotype that does not fully recapitulate the effects observed with isoform-specific targeting. This underscores the importance of considering isoform-specific functions of NOSs in HU-mediated stress responses.

In both HEL92.1.7 and mERP cells, Nos3 modulation decreased HU-induced S-phase arrest. HU-induced NOS3 mediated S-phase accumulation by increasing the G_0_/G_1_ fraction. While an increase in G_0_/G_1_ can reflect either cell cycle arrest or progression [26], a parallel decrease in ssDNA levels and increase in Ki67 expression suggest that the observed shift represents reduced replication stress and enhanced proliferation [27]. It should be noted that untreated HEL92.1.7 erythroleukemic cells exhibit a higher proportion of cells in the S-phase compared to mERPs, which predominantly reside in the G_0_/G1 phase, indicating higher basal replicative activity. These baseline differences likely influence the cell cycle redistribution observed with DPI treatment. In mERP cells, combined inhibition of NOS2 and NOS3 during HU treatment mirrored the NOS3-mediated S-to-G_0_/G_1_ accumulation and NOS2-mediated decrease in apoptosis, whereas HEL92.1.7 cells progressed through to G_2_/M, accompanied by increased late apoptosis. Another interesting hypothesis relates to their malignant versus healthy nature and the distinct redox states of HEL92.1.7 and mERP cells. Healthy mERPs are characterized by low basal reactive oxygen species (ROS) levels, making them more sensitive to NADPH oxidase inhibition and allowing the restoration of cell proliferation and survival. Malignant cells, however, exhibit higher basal ROS levels and impaired DNA repair [28]; thus, DPI treatment perturbed redox homeostasis and exacerbated replication stress in HEL92.1.7 cells, contributing to increased apoptosis. Future studies should directly assess the roles of NOS isoforms, ROS, and HU in DNA damage and checkpoint responses to validate these hypotheses.

HU has been reported to attenuate the growth of early erythroid colonies in a dose-dependent manner, which is not influenced by the NOS inhibitor L-NAME in the presence of L-arginine, suggesting NOS independence [29]. In contrast, our results indicate that Nos3 contributes to erythropoiesis while suppressing myeloid lineage commitment. Moreover, Nos2-derived NO has been implicated in stress erythropoiesis in mice [30], whereas Nos1 contributes to erythropoietin-stimulated erythropoiesis [31]. Previous studies have highlighted isoform-specific roles of NO signaling in hematopoiesis. For example, NOS2-derived NO has been implicated in the promotion of myeloid differentiation [32], while the NOS1–NOS-interacting protein (NOSIP) complex has been shown to regulate granulopoiesis [33]. These findings support the concept that distinct NOS isoforms contribute to hematopoietic regulation in a context-dependent manner.

We provide evidence that Nos2 and Nos3 are essential for HU-induced protein nitrosylation in bone marrow, as knockout of either isoform attenuated the nitrosylation of a ~60 kDa protein band, suggesting their cooperative role. The ~60 kDa band likely corresponds to mitochondrial Hsp60, an established target of NOS2-mediated S nitrosylation [34] or the p65 subunit of NFκB [35]. For proteins around ~40 kDa, nitrosylation was reduced in Nos2- but not Nos3-deficient mice, indicating Nos2-specific targets or compartmentalized NO effects. Cytoskeletal protein β-actin (~42 kDa) is a well-known target of S nitrosylation [36]. This aligns with emerging knowledge that different NOS isoforms generate distinct sets of S-nitrosylated proteins [37].

Molecular docking simulations predicted that HU binds within the active site of NOS3 via hydrogen bond formation with residues critical for L-arginine positioning. However, in vitro enzymatic assays with recombinant NOS3 revealed that HU does not directly alter NO or citrulline production, indicating that HU binding to NOS3 does not directly stimulate enzymatic activity. Nevertheless, non-catalytic ligand binding may exert indirect or allosteric effects on NOS3 regulation, such as stabilizing specific enzyme conformations, influencing cofactor accessibility, or altering interactions with other proteins. HU enhances NOS3 activity through activation of AKT1, a kinase known to activate NOS3 [13,25], and inhibition of AKT1 abrogates HU-induced NOS3 phosphorylation. The delayed kinetics of NOS3 activation further support an indirect mechanism rather than interaction-mediated enzymatic activation.

A limitation of our study is the limited selectivity and potential off-target effects of the pharmacological inhibitors used to modulate NOS activity. CSD, commonly used as an inhibitor of NOS3 activity, has been reported to down-regulate the expression of inflammatory cytokines and NOS2 while increasing the heme oxygenase-1 activity [38]. Such pleiotropic actions may influence apoptotic signaling independently of NOS3 inhibition and could contribute to the observed decrease in late apoptosis following CSD treatment. Notably, we previously demonstrated that NOS2 depletion markedly reduces apoptosis in both human and murine erythroid cells, suggesting that indirect modulation of NOS2 signaling by CSD may partially account for this effect. Interestingly, NOS3 knockdown increased late apoptosis under HU treatment in contrast to the effect observed with CSD. This discrepancy may also reflect the difference between complete loss of the NOS3 protein (including scaffolding or regulatory functions) and inhibition of its enzymatic activity. Similarly, DPI is a broad flavoprotein inhibitor that targets NADPH oxidases in addition to NOS isoforms [39]. Consequently, the cellular responses observed following DPI treatment likely reflect a broader disruption of flavoprotein-dependent redox signaling rather than selective inhibition of NOS2 or NOS3. The differential cell cycle and survival responses observed in malignant HEL92.1.7 and healthy mERP cells may arise from different sensitivity to replicative and oxidative stress. Importantly, inhibitor concentrations were determined through dose- and time-dependent optimization and were consistent with previously published ranges (Supplementary Table S1), which may help minimize but cannot fully exclude off-target effects. Taken together, these observations suggest that NOS-dependent redox signaling contributes to the cellular response to HU, while the precise contribution of individual NOS isoforms should be interpreted with caution, considering the pharmacological limitations. Furthermore, a relatively small sample size used in animal experiments may limit statistical power and the ability to detect more subtle biological effects. The present study did not include cell cycle progression analysis or identify specific S-nitrosylated proteins. While such analyses would provide deeper mechanistic insight, they are beyond the scope of the current work and represent important directions for future investigations.

## 5. Conclusions

Our study identified NOS3 as a key mediator of HU-induced replication stress and S-phase arrest in erythroid cells. HU enhanced NOS3 expression and activity, contributing to NO production and protein nitrosylation. In addition, NOS3 modulates apoptosis by reducing early apoptotic events while promoting late apoptosis under HU treatment. These dual effects suggest that NOS3 fine-tunes the balance of erythroid cell survival during HU treatment. Moreover, combined NOS2/NOS3 mediated HU-induced cell cycle arrest and apoptosis, suggesting their complementary role. Understanding this dual regulation may offer novel insights for optimizing HU therapy in hematologic malignancies, such as by enhancing NOS activity to potentiate HU efficacy or developing HU derivatives with improved NOS-activating capacities.

## Data Availability

The original contributions presented in this study are included in the article/Supplementary Material. Further inquiries can be directed to the corresponding author.

## Supplementary Materials

The following supporting information can be downloaded at https://www.mdpi.com/article/10.3390/antiox15040435/s1. Figure S1: Hydroxyurea induces NOS3 expression and activity in HEL92.1.7 cells; Figure S2: NOS3 deletion or inhibition shifts cells from S to G0/G1 phase and regulates apoptosis under hydroxyurea treatment; Figure S3: Nos3 deficiency impairs hydroxyurea-induced protein nitrosylation and alters hematopoietic lineage commitment in vivo; Figure S4: In vivo NOS3 depletion or inhibition impairs hydroxyurea-mediated S-phase blockage and alters apoptosis; Figure S5: Dual NOS2/NOS3 inhibition impairs hydroxyurea-induced proliferation block in erythroid cells; Figure S6: Dual NOS2/NOS3 inhibition impairs hydroxyurea-induced apoptosis of erythroid cells in a context-dependent manner; Table S1:Nitric oxide synthase (NOS) inhibitors’ affinity and potency; Table S2:An overview of the effects of NOS depletion and inhibition on HU response. References [40,41,42,43,44,45,46,47,48,49] are cited in the Supplementary Material.

## Abbreviations

The following abbreviations are used in this manuscript:

AKT	RAC-alpha serine/threonine protein kinase
BFU-E	Burst-forming unit—erythroid
Cas3	Caspase 3
CD71	Cluster of Differentiation 71
CFU-E	Colony-forming unit—erythroid
CFU-GM	Colony-forming unit—granulocyte/macrophage progenitors
CSD	Caveolin-1 scaffolding domain peptide
DPI	Diphenyleneiodonium chloride
FACS	Fluorescence-activated cell sorting
GFP	Green fluorescent protein
HU	Hydroxyurea
Ki	Inhibition constant
Ki67	Antigen Kiel 67
L-NAME	NG-nitro-L-arginine-methyl ester
L-NMMA	N(G)-monomethyl-L-arginine
L-NNA	NG-nitro-L-arginine
mERPs	Mouse erythroid progenitors
NFκB	Nuclear factor kappa-light-chain-enhancer of activated B cells
NO	Nitric oxide
NOS	Nitric oxide synthase
NOS1	Neuronal NOS
NOS2	Inducible NOS
Nos3^-/-^	Nos3 knockout mice, Nos3 null mice
NOS3	Endothelial NOS
NOS3_kd_	NOS3 knockdown
NO_x_	Nitrite and nitrate
NOX	(NADPH oxidase)
NOSIP	Nitric oxide-interacting protein
PAI	Proliferation to apoptosis index
PBS	Phosphate-buffered saline
PI	Propidium iodide
PKA	cAMP-dependent protein kinase
RBCs	Red blood cells
ROS	Reactive oxygen species
Rg	Radius of gyration
RMSD	Root Mean Square Deviation
RMSF	Root Mean Square Fluctuation
SEM	Standard error of mean
shRNA	Short hairpin RNA
ssDNA	Single-stranded DNA
Ter119	Surface protein associated with glycophorin-A
UPS	Uprosertib
WT	Wild type
ΔE_elec_	Energy of electrostatic interactions
ΔG_binding_	Gibbs free binding energy
ΔG_nonpolar_	Non-electrostatic nonpolar free energy
