# Supplementary material for: Endothelial Nitric Oxide Synthase-Dependent Mechanism of Hydroxyurea-Induced S-Phase Arrest in Erythroid Cells
Source: Antioxidants (Basel). 2026 Mar 31;15(4):435. doi: 10.3390/antiox15040435 (PMC13113100; doi:10.3390/antiox15040435)
Supplement: Supplementary file 1 [file antioxidants-15-00435-s001.zip › antioxidants-4186782-supplementary.pdf]

Supplementary Information File

Endothelial nitric oxide synthase-dependent mechanism of hydroxyurea-induced S phase arrest in erythroid cells

Teodora Dragojević[1], Dragoslava Đikić[1], Slavko Mojsilović[2], Miloš Lazarević[3], Dejan Milenković[4], Olivera Mitrović Ajtić[1], Emilija Živković[1], Miloš Diklić[1], Tijana Subotički[1], Juan F. Santibanez[1], Vladan P. Čokić[1], and Milica Vukotić[1]

Affiliations:

[1]Department of Molecular Oncology, Institute for Medical Research, National Institute of the Republic of Serbia, University of Belgrade, Belgrade, Serbia

[2]Department of hematology and stem cells, Institute for Medical Research, National Institute of the Republic of Serbia, University of Belgrade, Belgrade, Serbia

[3] Department for Human Genetics, Implantology Research Center, Faculty of Dentistry, University of Belgrade, Belgrade, Serbia

[4]Institute for Information Technologies, University of Kragujevac, Kragujevac, Serbia

Corresponding author:

Milica Vukotić

Department of Molecular Oncology

Institute for Medical Research, University of Belgrade

Dr. Subotica 4, 11129 Belgrade, Serbia

Phone: +381112684484

Fax: +381112643691

E-mail: milica.tosic@imi.bg.ac.rs

Supplementary Figure S1.

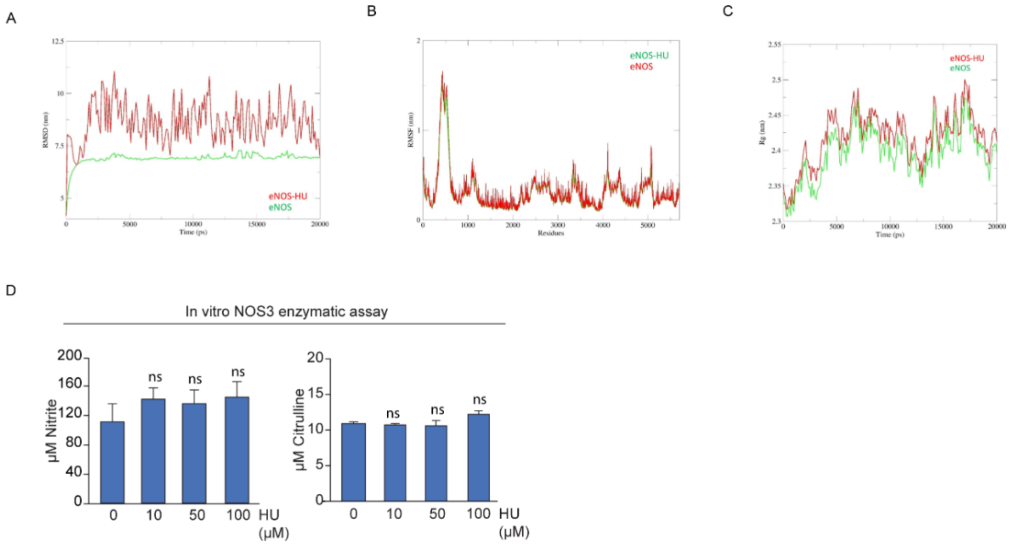

Supplementary Figure S1. Hydroxyurea induces NOS3 expression and activity in HEL92.1.7 cells. Molecular dynamic analyses showing: **A)** Root Mean Square Deviation (RMSD) of C–Cα–N backbone vs. simulation time for NOS3 in complex with and without HU during 20 ns, **B)** Root Mean Square Fluctuation (RMSF) values of NOS3-HU complex plotted against residue numbers, and **C)** Radius of gyration (Rg) plots of NOS3 receptor with and without HU in active sites during 20 ns. **D)** Concentration of nitrite or citrulline measured after *in vitro* NOS3 enzymatic assay with indicated concentrations of HU. $n = 3$; mean + SEM, ns- non-significant vs. control.

Supplementary Figure S2.

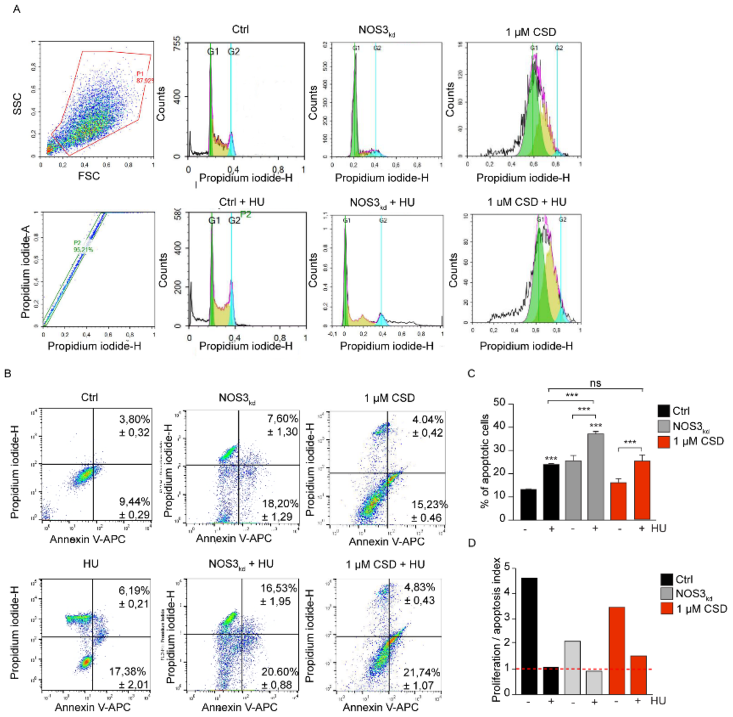

Supplementary Figure S2. NOS3 deletion or inhibition shifts cells from S to G0/G1 phase and regulates apoptosis under hydroxyurea treatment. **A)** Example of gating for cell cycle analysis in $NOS3_{kd}$ and CSD-inhibited (1 µM) HEL92.1.7 cells treated with HU or vehicle. Debris and doublets were excluded, and the cell cycle distribution was determined based on the incorporation of PI. **B)** Example of gating for Annexin V/PI apoptotic assay in $NOS3_{kd}$ and CSD-inhibited (1 µM) HEL92.1.7 cells treated with HU or vehicle. The percentage of apoptotic cells was calculated as the sum of the upper and bottom right quadrants. **C)** The percentage of Annexin V+/PI+ apoptotic cells. **D)** Proliferation to apoptosis index (PAI) representing relative fractions of $Ki67^{+}$ actively proliferating population and Annexin $V^{+}/PI^{\pm}$ total apoptotic cells (PAI < 1 – apoptosis, PAI >1 – proliferation). $n = 3$; mean + SEM, ***$p < 0.001$ vs. control or as indicated; ns- non-significant.

Supplementary Figure S3.

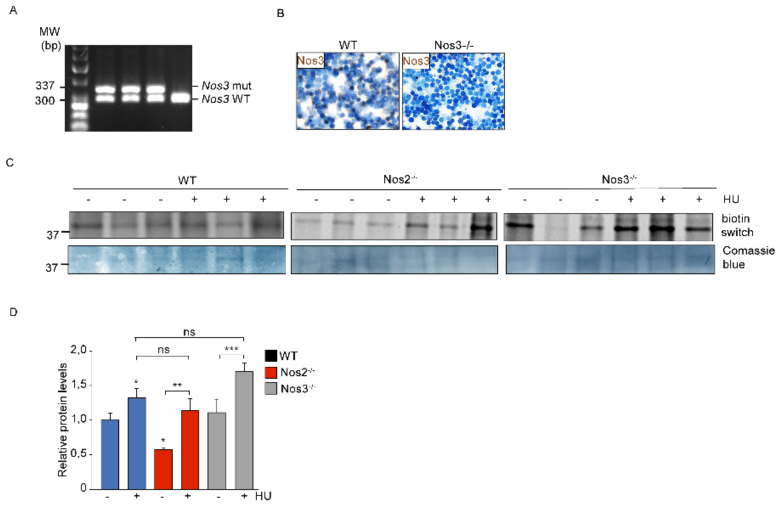

Supplementary Figure S3. Nos3 deficiency impairs hydroxyurea-induced protein nitrosylation and alters hematopoietic lineage commitment in vivo. **A)** Genotyping of Nos3$^{-/-}$ mice. Wild-type *Nos3* allele (*Nos3* WT) at 300 bp and mutated *Nos3* (Nos3 mut) allele at 337 bp. **B)** Immunocytochemistry for Nos3 protein in mERP isolated from WT mice treated with HU or vehicle. **C)** Nitrosylation of proteins at 40kDa in bone marrow of WT, Nos2$^{-/-}$, and Nos3$^{-/-}$ mice treated with HU or vehicle was visualized using the Biotin switch kit, while Coomassie blue staining was used to detect total proteins. **D)** Quantification of band intensity with Coomassie blue used as a loading control and normalized to control. $n = 3$; mean + SEM, *$p < 0.05$, **$p < 0.01$, ***$p < 0.001$ vs. control (Ctrl) or as indicated; ns- non-significant.

Supplementary Figure S4.

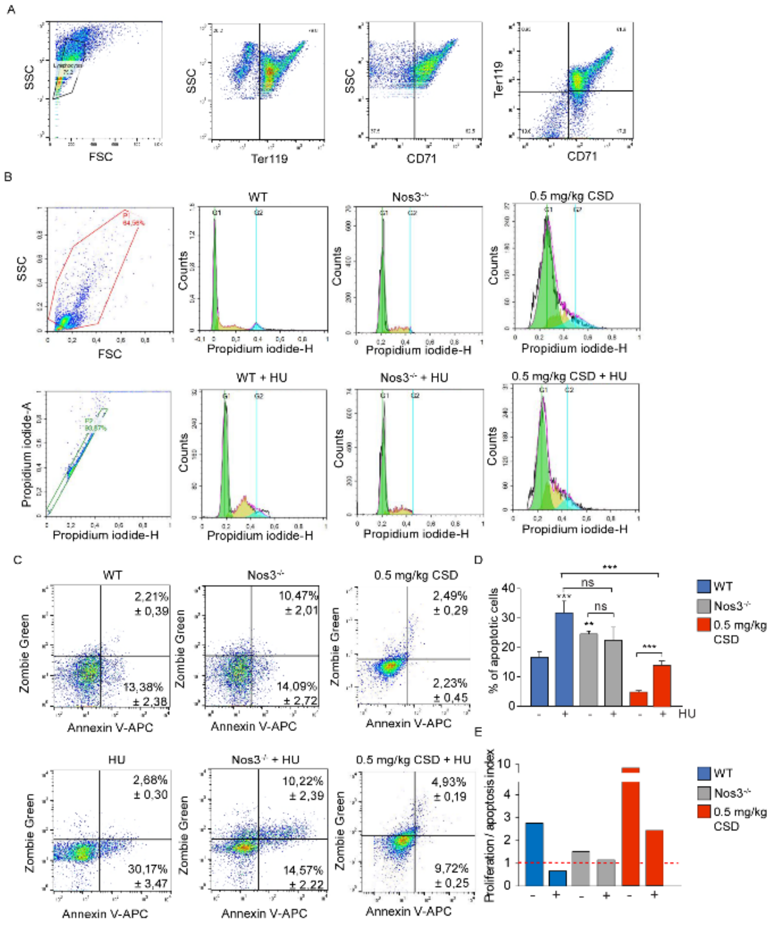

Supplementary Figure S4. In vivo NOS3 depletion or inhibition impairs hydroxyurea-mediated S-phase blockage and alters apoptosis. **A)** Purity of mouse erythroid progenitors (mERP) isolated from bone marrow by immunomagnetic cell separation was verified by flow cytometry. An example of gating for CD71- and Ter119-positive cells is shown, where the upper-right quadrant represents the double-

positive population. **B)** Example of gating for cell cycle analysis in Nos3$^{-/-}$ and wild-type (WT) mERP cells treated with HU or vehicle, or WT mERP treated with 0,5 mg/kg CSD. **C)** Example of gating for Annexin V/PI apoptotic assay in Nos3$^{-/-}$ and WT mERP cells treated with HU or vehicle, or WT mERP treated 0,5 mg/kg CSD. The percentage of apoptotic cells was calculated as the sum of the upper and bottom right quadrants. **D)** The percentage of Annexin V+/PI+ apoptotic cells. **E)** Proliferation to apoptosis index (PAI) representing relative fractions of Ki67^{+} actively proliferating population and Annexin V^{+}/PI$^{\pm}$ total apoptotic cells (PAI < 1 – apoptosis, PAI >1 – proliferation). $n = 3$; mean + SEM, **$p < 0.01$,***$p < 0.001$ vs. WT or as indicated; ns- non-significant.

Supplementary Figure S5.

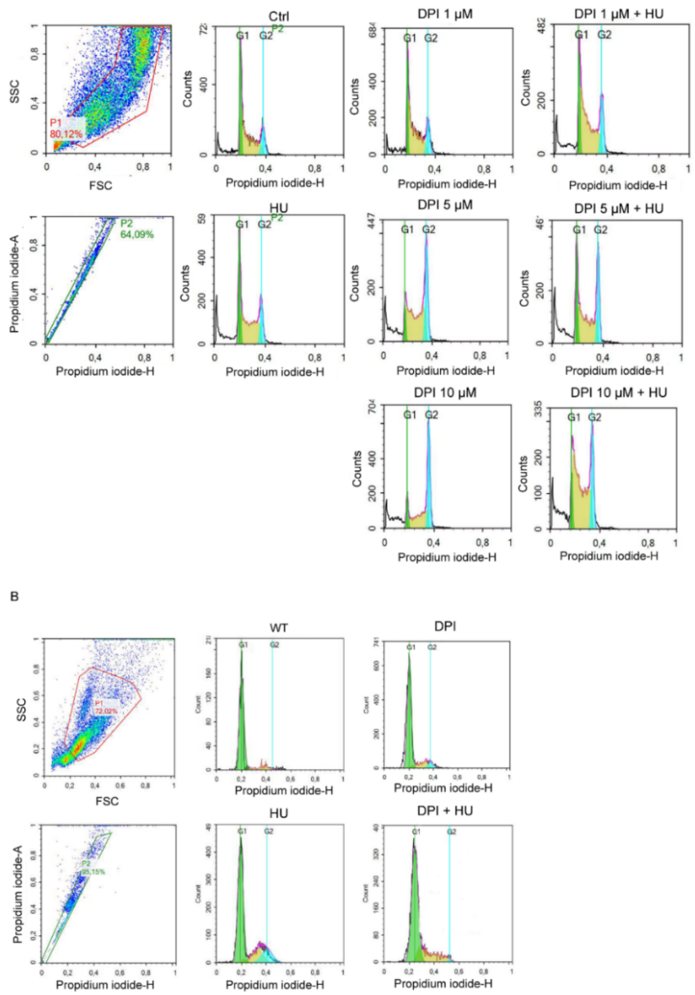

Supplementary Figure S5. Dual NOS2/NOS3 inhibition impairs hydroxyurea-induced proliferation block in erythroid cells. Example of gating for cell cycle distribution upon PI staining in **A)** HEL92.1.7 cells or **B)** wild-type (WT) mERP cells treated with HU, DPI, or combination. $n = 3$.

Supplementary Figure S6.

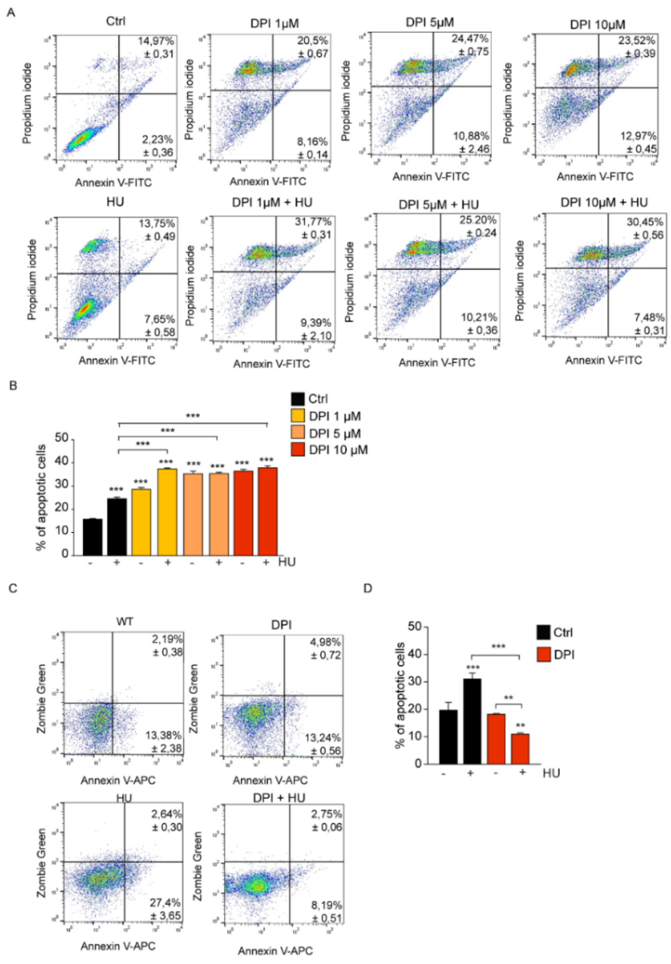

Supplementary Figure S6. Dual NOS2/NOS3 inhibition impairs hydroxyurea-induced apoptosis of erythroid cells in a context-dependent manner. Example of gating for Annexin V/PI apoptotic assay in **A)** HEL92.1.7 cells and **B)** the percentage of Annexin V+/PI+ apoptotic cells. Example of gating for Annexin V/PI apoptotic assay in **C)** Wild-type (WT) mERP cells treated with HU, DPI, or

combination. **D)** The percentage of apoptotic cells was calculated as the sum of the upper and bottom right quadrants. $n = 3$; mean + SEM, $^{**}p < 0.01$, $^{***}p < 0.001$ vs. control (Ctrl)/WT or as indicated.

Supplementary Table S1.

NOS Inhibitors			NOS1		NOS2		NOS3		[μM] in cell assay		[mg/kg] in mice	
**Name**	**Specificity**		**Ki**	**IC_{50}**	**Ki**	**IC_{50}**	**Ki**	**IC_{50}**	**Current**	**Literature**	**Current**	**Literature**
**1400W**	**NOS 2**	**[NI]**	2 μM[40]	154±78 μM[41]	7 nM[40]	0.8±0.3 μM[40]	50 μM[40]	744±52 μM[41]	1, 10, 100	10[42], 100[43]	20	10[44], 20[45]
		**assay**	cell-free	tissue	cell-free	tissue	cell-free	tissue				
		**activity**	citrulline production	cGMP production	citrulline production	aortic ring contraction	citrulline production	cGMP production				
**CSD**	**NOS 3**	**[NI]**		1.8 μM[46]				49 nM[47]	5	10[18]		
		**assay**		cell-free				cell-free				
		**activity**		NO production and NADPH oxidation				fluorescence polarization experiment				
**DPI**	**NOS 2,3**	**[NI]**				30 nM[19]		300 nM[48]	1, 5, 10	5[49], 1-10[19]	1	1[23]
		**assay**				cell		tissue				
		**activity**				NO production		aortic ring contraction				

Supplementary Table S1. Nitric oxide synthase (NOS) inhibitors' affinity and potency

IC_{50} (Half Maximal Inhibitory Concentration - lower the IC_{50} - more potent the inhibitor); K_i (Inhibition Constant – lower the K_i - stronger the binding (higher affinity)). DPI (Diphenyleneiodonium chloride), 1400W (N-3-(Aminomethyl), Caveolin-1 scaffolding domain peptide (CSD), concentration of NOS inhibitor [NI].

Supplementary Table S2.

HEL 92.1.7 cells	Proliferation (Ki67)	Replication stress (S-phase arrest)	Replication stress / DNA damage (ssDNA)	Early apoptosis (Annexin V+/PI-)	Late apoptosis (Annexin V+/PI+)
**$NOS3_{kd}$**	+	-	-	ns	+
**L-NAME**	+	+	-	+	+
**1400W**	+	ns	-	-	-
**CSD**	+	-	-	ns	ns
**DPI**	+	-	-	ns	+

Supplementary Table S2. An overview of the effects of NOS depletion and inhibition on HU response. The effects of NOS3 knock-down ($NOS3_{kd}$), and NOS inhibition [by pan-NOS inhibitor NG-nitro-L-arginine-methyl ester (L-NAME), NOS2-specific inhibitor 1400W, NOS3 inhibitor caveolin 1 scaffolding domain peptide (CSD), and NOX/NOS inhibitor diphenyleneiodonium chloride (DPI)] on HU-mediated proliferation inhibition, S-phase arrest, and DNA damage and apoptosis induction in erythroleukemic HEL92.1.7 cells. Plus – increased; minus – decreased; ns – non-significant.
